# Exploring Gender Disparities in Emergency Department Utilization: A Comprehensive Comparative Analysis of the Frequency of Female Versus Male Emergency Department Visits

**DOI:** 10.7759/cureus.68066

**Published:** 2024-08-28

**Authors:** Ali A Aalam, Nofel Iftikhar, Naveen Baskaran, Adnan Bhat

**Affiliations:** 1 Epidemiology, University of Florida, Gainesville, USA; 2 Biology, University of Florida, Gainesville, USA; 3 Medicine, University of Florida College of Medicine, Gainesville, USA

**Keywords:** 2012-2021, united states of america, neds, emergency department utilization, gender disparities

## Abstract

Background

The emergency department (ED) serves as the front line for emergency patient care, a unique limbo between the realms of generalist care and specialists and outpatient and inpatient care. As the trend of utilizing emergency healthcare services has escalated in the past 10 years, it becomes interesting to look at the patient characteristics of those who have utilized the ED. This analysis can guide the operation of impartial care for all ED patients to ensure equal access and discourage disparity based on the patient’s sex.

Methods

This cross-sectional analysis seeks to examine differences in the frequency of patient visits to the ED in the United States (US) based on the patient's sex. The study utilizes data from the period 2012 to 2021, which was derived from the Nationwide Emergency Department Sample (NEDS).

Results

Analyzing both the rates of female and male visits to the ED on a year-by-year basis within the study period, it was observed that a higher proportion of females visit the ED, and, in general, both females and males have visited the ED less frequently in recent years. However, while the female sex has continued to represent the majority of ED patients, the gap between the majority female population and the minority male population has decreased, especially dramatically, in recent years. Additionally, using the compiled data and a linear regression model, predicted values were forecasted to continue this trend, with a general decrease in ED visits and a narrowing of the gap between females and males from 2022 to 2032.

Conclusion

As discussion and policy surrounding healthcare equity, primarily based on gender, becomes a more relevant topic, it is essential to discuss and analyze data to base new healthcare policy to ensure healthcare equality in all fields of medicine, including the ED and beyond. This study aims to identify and compare the difference in the frequency of ED visits between males and females to improve patient outcomes and further discussion regarding healthcare equity.

## Introduction

The emergency department (ED) serves as the front line for general patient care, a unique limbo between the realms of generalist care and specialists and outpatient and inpatient care [1-2]. As the general usage of emergency healthcare services has, in general, increased over the past decade, it is essential to view the demographic characteristics of individuals visiting the ED to elucidate potential discrepancies in utilization and ensure health equity for all ED [3-6]. While these demographics can be divided into several subcategories, including those with specific medical diagnoses (including visits based on chronic diseases and acute injuries) and ethnic demographics (including country of origin and self-defined racial or ethnic identity), the classification of interest in this study is sex [7-8].

Especially recently, as interest in sex differences in patient care becomes a more studied and relevant topic, it is important that, as a healthcare community, it is ensured that patient experience and outcomes are independent of sex [9-10]. This idea runs parallel to recent discussions and the implementation of new health equity policies, which are a monumental step in health equality for all [11-12].

Perhaps most important to implementing these new policies regarding health equity is understanding healthcare data regarding the frequency of visits and, most importantly, the difference in frequency of visits between males and females [13]. These data can help serve as a baseline for additional research and healthcare policy. Since the ED represents such an essential and utilized first step in the healthcare system, starting with data from the Nationwide Emergency Department Sample (NEDS) database, a tool developed by the Healthcare Cost and Utilization Project (HCUP), was a critical preliminary option [14].

In exploring trends related to the frequency of visits, much can be said regarding other issues and topics relevant to emergency medicine and healthcare in general. For example, analyzing sex trends may give rise to discussion or policy regarding male and female staff proportions in EDs and patient preference surrounding the physician who treats them [15-16]. Additionally, analyzing visit frequency naturally gives rise to discussions surrounding overcrowding and ED utilization [17-18]. Naturally, a reason for conducting this study is the opportunities it presents regarding the analysis of other variables related to healthcare discussion, which is an exciting prospect.

This study aims to investigate the trends in ED utilization among female and male patients using a decade-long dataset extracted from the NEDS database spanning from 2012 to 2021.

## Materials and methods

A comparative retrospective analysis was conducted to contrast the healthcare utilization patterns of female and male patients in the ED settings. Utilizing the NEDS database, the largest all-payer publicly available ED database in the US, and a tool developed by the HCUP, data were collected regarding the frequency of male and female ED visits in the period 2012 to 2021. In addition to sex, information regarding gender, insurance type, and age was collected for a potential comparative analysis, which indicated the mean age for all the ED patients from 2012 to 2021 was 41 years old, and the most common insurance type they had was Medicaid. The data in NEDS are de-identified for public use and safeguarded through data use agreements. Consequently, analyses and publications utilizing NEDS data are exempt from institutional review board (IRB) review.

Descriptive statistics were employed to elucidate temporal trends in ED visits, focusing on the absolute visits and percentage distribution of female versus male patients relative to total ED visits over the study period on a year-by-year basis. From 2012 to 2021, the mean age for a patient visiting the ED was calculated by dividing the sum of the ages of all ED patients ranging from 0 to 90 years by the number of all the patients coming to the ED. The insurance status (Medicare, Medicaid, and Private Insurance) for each patient coming to ED each year from 2012 to 2021 helped in identifying the most predominant insurance status for an ED patient. The relative rate of male patients visiting the ED in a specific year was calculated by dividing the total number of male patients seen that year in the ED by the total number of ED visits. The relative rate of female patients visiting the ED in a specific year was calculated by dividing the total number of female patients seen that year in the ED by the total number of ED visits. Subsequently, utilizing a linear regression model, the forecasted values of male and female visits to the ED were calculated within the period from 2022 to 2032. Additionally, to better define larger data points, several descriptive values were calculated; these included the mean, the median, the q1 value, the q3 value, and a 95% confidence interval (CI). Using these values and analyzing general trends, a holistic view of the differences in ED visit frequencies based on sex was elucidated.

## Results

First, analyzing the frequency of ED visits by males, in the study period from 2012 to 2021, a total of 618.41 million men visited the ED, with an average of 61.84 million annually, 95% CI of (59.76 million annually, 63.93 million annually), q1 = 60 million visits annually, median = 62.75 million visits annually, and q3= 64.5 million visits annually. The highest frequency of males visiting the ED in 2017 was 64,576,069 visits. Conversely, the lowest frequency of males was seen in the ED in 2020, with a total of 56,899,023 visits. Overall, analyzing the trend of male visits to the ED from 2012 to 2019, an overall positive trend was seen, with a drastic downward turn being seen in 2020, most likely attributed to the COVID-19 pandemic. Following 2020, into the end of the study period, an upturn in male visits to the ED was observed in 2021.

Although the overall number of male patients visiting the ED followed a unimodal trend, featuring lower frequencies at the beginning and end of the study period, the rate of males visiting the ED relative to total ED visits followed a positive trend. Although remaining relatively stagnant from 2012 (44.5%) to 2018 (44.8%), only fluctuating by 0.3% over the seven years, 2019 saw an increase in the proportion with males making up 45.1% of all ED patients, this proportion increasing further in 2020 (46.2%), and declining by the slightest margin in 2021 (46%).

Then, analyzing the frequency of ED visits by females, in the study period from 2012 to 2021, a total of 758.81 million females visited the ED, with an average of 77.68 million visits annually, 95% CI of (72.36 million visits annually, 79.41 million visits annually), q1 = 74.51 million visits, median = 76.3 million annually, and q3 = 79.30 million visits annually. The highest frequency of females presented to the ED in 2016, with a total of 80,367,788 visits. Contrarily, the lowest frequency of females presented to the ED in 2020, with a total of 66,369,143 visits. Overall, analyzing the trend of female visits to the ED from 2012 to 2017, an overall positive trend was observed with a year-after-year increase. Following 2017, from 2018 to 2020, a negative trend was observed, with a drastic dip seen in 2020, an anomaly most likely attributed to the COVID-19 pandemic. Following 2020, into the end of the study period, an upturn in female visits to the ED was seen in 2021, with a total of 68,601,012 visits.

Akin to the overall trends in male visits to the ED, female visit frequency followed a unimodal frequency peaking toward the middle of the study period. However, in contrast with the analysis regarding male visits, the female visit rate relative to total ED visits solely decreased. In 2013, the relative rate featured a high of 55.5%, remaining relatively stable through 2018 (55.2%), fluctuating only 0.3%. Following 2018, however, the relative rate dipped in 2019 (54.9%) and then plummeted to a low in 2020 (53.8%) before recovering slightly in 2021 (54%).

The frequency of both total male and female visits to the ED, annual ED visits, and male and female ED visits relative to total ED visits on an annual basis are represented in Table 1.

**Table 1:** Table showcasing values for total ED patients, male ED patients, and female ED patients, along with the rate of male and female patients, relative to total ED patients on an annual basis in the US from 2012 to 2021

Year	Total Number of ED Visits	Male ED Patients	Female ED Patients
2012	134,399,179	59,876,517 (44.5 %)	74,512,661 (55.4%)
2013	134,869,015	60,049,843 (44.5%)	74,814,441 (55.5%)
2014	137,807,901	61,320,111 (44.5%)	76,481,534 (55.5%)
2015	143,469,670	64,201,941 (44.8%)	79,249,348 (55.2%)
2016	144,842,742	64,453,186 (44.5%)	80,367,788 (55.4%)
2017	144,814,803	64,576,069 (44.6%)	80,230,557 (55.4%)
2018	143,454,430	64,150,812 (44.8%)	79,295,835 (55.2%)
2019	143,432,284	64,539,409 (45.1%)	78,881,514 (54.9%)
2020	123,278,165	56,899,023 (46.2%)	66,369,143 (53.8%)
2021	126,968,321	58,352,350 (46%)	68,601,012 (54%)

Comparing the frequency of males and females, females were seen in the ED with a higher frequency every year of the study period when compared to males. Quantifying this, on average, 14 million more females were seen in the ED annually than males; this discrepancy was the largest in 2016 year, with 15,914,602 more females being seen, and the smallest in 2020, with 9,470,120 more females being seen in the ED. As mentioned, the trends in male and female ED visit frequency also differ in their positive and negative tendencies. As reflected in the data presented earlier, male ED visit frequency generally increased from 2012 to 2019, plummeting in 2020 because of the COVID-19 pandemic and then recovering slightly in 2021. The female ED visit frequency is somewhat different. Analyzing the trend, there was a positive increase in visit frequency from 2012 to 2017, a slight decrease from 2018 to 2019, then a sudden decrease in 2020, attributed to the COVID-19 pandemic, and then a slight recovery in 2021.

While both male and female visit frequency has decreased over the study period, contributing to the overall decrease in the total number of ED visits, the once large discrepancy between male and female utilization of the ED has narrowed. Especially when analyzing sex-specific visit rates relative to total ED visits, this growing gap becomes obvious. While toward the beginning of the study period, from 2012 to 2018, the average relative rate discrepancy between female and male visits to the ED was 10.77%, favoring females. However, this divergence diminished between 2019 and 2021, with an average difference of 8.47%

**Figure 1:**
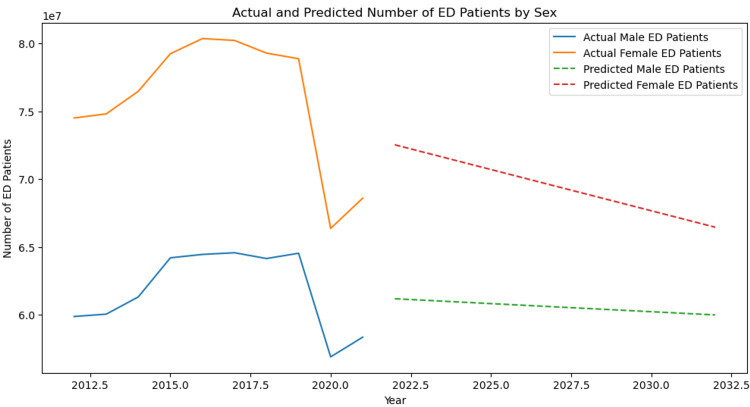
Line graph showing the frequencies for total male ED visits and female ED visits in the US from 2012 to 2021 and forecasted values for male ED visits and female ED visits within the predictive period from 2022 to 2032 using a linear regression model

Compiling the data from 2012 to 2021, a linear regression model was processed to predict male and female ED visit rates from 2022 to 2032. The predicted values for both sexes correlated with currently observed trends, as the value of both male and female visits to the ED are forecasted to decrease with the female rate decreasing more rapidly and the discrepancy between visit rates for the two sexes further shrinking. The forecasted values, using a linear regression model, from 2022 to 2032, for total ED visits, male visits to the ED, and female visits to the ED are shown in Table 2, and a graphical representation of forecasted values, from 2022 to 2032, is depicted in Figure 1.

**Table 2:** Predicted values for total ED visits, male ED visits, and female ED visits in the US from 2022 to 2032 using a linear regression model

Year	Total Number of ED Visits	Male ED Patients	Female ED Patients
2022	133,734,800	61,185,017	72,536,390
2023	133,007,700	61,065,579	71,928,391
2024	132,280,700	60,946,141	71,320,392
2025	131,553,600	60,826,703	70,712,393
2026	130,826,600	60,707,265	70,104,394
2027	130,099,500	60,587,827	69,496,396
2028	129,372,400	60,468,390	68,888,397
2029	128,645,400	60,348,952	68,280,398
2030	127,918,300	60,229,514	67,672,399
2031	127,191,200	60,110,076	60,110,076
2032	126,464,200	59,990,638	66,456,402

## Discussion

The ED visit frequency trends, in both males and females, reveal a concerning trend of declining ED visits, which puts individuals at risk for the underutilization of medical services, especially when compared to the data of previous decades. While this could be a result of numerous other external factors, including improvements in preventive care and outpatient services, even a partial true underutilization of emergency medical services bears intensive analysis and scrutinization.

This can most readily be seen when analyzing the trend regarding female visit frequency to the ED. Within the study period, the most females visited the ED in 2017, with a staggering 80.3 million visits. Since 2017, this value has steadily decreased, hitting a low in 2020 with only 66.3 million visits. Although, as previously mentioned, the frequency of visits in 2020 was unnaturally low due to the COVID-19 pandemic, it was only able to recover slightly into 2021, with a total of 68.6 million visits made in the entire year. The fluctuation in female visits to the ED represents a stark contrast when compared with the trends observed in the male frequency of ED visits. In recent years, the most common reasons for female visits to the ED have been abnormal bleeding, pelvic pain, gynecological abnormalities, an assortment of pregnancy-related issues, and general non-specific injuries. While these issues are not exclusive to women, they are observed more in women, especially when compared to men [19-20].

As mentioned, the frequency of male visits to the ED remained relatively stable, apart from the sudden downturn in visits in 2020, induced by the COVID-19 pandemic, ranging only from approximately 60 to 65 million visits annually from 2012 to 2019, even reaching a constant of approximately 64 million visits from 2015 to 2018. For men, the most common complaints that led to a visit to the ED included abdominal pain, chest pain, sprains, cuts, contusions, and back pain, a set of complaints especially interesting as they bear almost no crossover with the most common complaints of women reporting to the ED [21-22].

While females continue to represent a higher proportion of ED visits compared to males, the decreasing difference in the number of visits between the two sexes underscores a concerning disparity in healthcare access and utilization [23]. Further analysis and study on the matter may yield highly relevant information as to the causes of the narrowing gap. Additionally, while the tail end of the data used in the study was collected in 2021, a year, especially in healthcare, still marred by the effects of the COVID-19 pandemic, it would be captivating to see whether the lower frequency of visits in 2021 was due to the lingering effects of the pandemic or a genuine downturn in utilization of the ED. Although the COVID-19 pandemic may have played a role in the lower visit frequencies seen in 2020 and 2021, the observed trends were already in motion as early as 2019, insinuating a true decrease in visit frequency, one which is more apparent in the decreasing rates of female visits to the ED. In addition to COVID-19, downturns in ED utilization may have been spurred by a number of other factors, including but not limited to healthcare policy changes, insurance-related legislation, and public health education. Future research should analyze the impact of these specific variables in more detail in an effort to produce a comprehensive analysis of ED utilization. Furthermore, efforts should be taken currently to address the current disparities present in the ED as policymakers should focus on promoting access to services, fostering sex-inclusive spaces, and optimizing both patient-care outcomes and the patient experience.

Limitation

Despite the numerous insights yielded by the study concerning the comparative frequencies of ED visits of male and female patients in the US in the period from 2012 to 2021, this study features limitations that bear mentioning. First, the retrospective nature of this study makes it impossible to discern definite causal relationships. Furthermore, since this study encompasses dates affected by the COVID-19 pandemic, which featured a large underutilization of the healthcare system, unrepresentative values may have been reported in 2020 and 2021, skewing the overall analysis and the predictive linear regression model values. Additionally, the predictions made were solely linear, a model that fails to capture more elaborate changes in healthcare usage. Finally, while the research separates the participants based on sex, it does not present results regarding non-binary and individuals self-identifying as trans or gender fluid, an important scope of study in the field of health equity and one that future studies should analyze. These limitations indicate that while this study has uncovered basic information regarding the sex-based differences in the utilization of ED services, additional investigations involving more specific and diverse information are required to comprehend the contributing factors to these trends as well as to provide relevant healthcare policies that will help in the achievement of sex equality.

## Conclusions

This study highlighted significant sex disparities in ED utilization rates. The data utilized in this study were collected from the NEDS database and then analyzed and compiled to better observe trends in the frequency of visits of males and females.

Regarding the rate of both sexes, a decrease in visits was seen over the study period. However, the rate, relative to total ED visits on an annual basis, of females decreased more significantly than the relative rate of males. Despite the persistent higher representation of females in ED settings, the diminishing trend raises concerns regarding equitable access to timely and appropriate healthcare services. This narrowing gap in ED utilization between the sexes may highlight factors regarding health inequity. Efforts to address these disparities should encompass targeted interventions aimed at enhancing healthcare access, promoting preventative care strategies, and fostering sex-sensitive healthcare delivery models to ensure equitable healthcare outcomes for all individuals. Further research should seek to pinpoint the factors surrounding the drop in female visits to the ED observed in this dataset.

## References

[1] Mackie D (2019). Role of the emergency department. Emerg Med Australas.

[2] Lim ME, Worster A, Goeree R, Tarride JÉ (2013). Simulating an emergency department: the importance of modeling the interactions between physicians and delegates in a discrete event simulation. BMC Med Inform Decis Mak.

[3] Hooker EA, Mallow PJ, Oglesby MM (2019). Characteristics and trends of emergency department visits in the United States (2010-2014). J Emerg Med.

[4] Latham LP, Ackroyd-Stolarz S (2014). Emergency department utilization by older adults: a descriptive study. Can Geriatr J.

[5] Burns TR (2017). Contributing factors of frequent use of the emergency department: a synthesis. Int Emerg Nurs.

[6] Northington WE, Brice JH, Zou B (2005). Use of an emergency department by nonurgent patients. Am J Emerg Med.

[7] Perkoff GT, Anderson M (1970). Relationship between demographic characteristics, patient's chief complaint, and medical care destination in an emergency room. Med Care.

[8] Hunt KA, Weber EJ, Showstack JA, Colby DC, Callaham ML (2006). Characteristics of frequent users of emergency departments. Ann Emerg Med.

[9] Azad AD, Charles AG, Ding Q, Trickey AW, Wren SM (2020). The gender gap and healthcare: associations between gender roles and factors affecting healthcare access in Central Malawi, June-August 2017. Arch Public Health.

[10] Teunissen TA, Rotink ME, Lagro-Janssen AL (2016). Gender differences in quality of care experiences during hospital stay: a contribution to patient-centered healthcare for both men and women. Patient Educ Couns.

[11] Braveman P (2006). Health disparities and health equity: concepts and measurement. Annu Rev Public Health.

[12] National Academies of Sciences, Engineering Engineering, and Medicine, Health and Medicine Division, Board on Population Health and Public Health Practice, Committee on Community-Based Solutions to Promote Health Equity in the United States (2017). Communities in Action: Pathways to Health Equity. https://www.ncbi.nlm.nih.gov/books/NBK425853/.

[13] Asiskovitch S (2010). Gender and health outcomes: the impact of healthcare systems and their financing on life expectancies of women and men. Soc Sci Med.

[14] (2024). NEDS Overview. http://hcup-us.ahrq.gov/nedsoverview.jsp.

[15] Huang KC, Lin YR, Syue YJ, Kung CT, Chiu IM, Li CJ (2018). Comparison of clinical practice in the emergency department: female versus male emergency physicians. Am J Med Sci.

[16] Nolen HA, Moore JX, Rodgers JB, Wang HE, Walter LA (2016). Patient preference for physician gender in the emergency department. Yale J Biol Med.

[17] Roberge D, Pineault R, Larouche D, Poirier LR (2010). The continuing saga of emergency room overcrowding: are we aiming at the right target?. Healthc Policy.

[18] Lombrail P, Vitoux-Brot C, Bourrillon A, Brodin M, De Pouvourville G (1997). Another look at emergency room overcrowding: accessibility of the health services and quality of care. Int J Qual Health Care.

[19] Verbrugge LM, Steiner RP (1984). Another look at physicians' treatment of men and women with common complaints. Sex Roles.

[20] Schwartz LR, Overton DT (1987). Emergency department complaints: a one-year analysis. Ann Emerg Med.

[21] Tam A, Lau F (2000). A three-year review of complaints in emergency department. Hong Kong J Emerg Med.

[22] Ooi SB (1997). Emergency department complaints: a ten-year review. Singapore Med J.

[23] Kruse MI, Bigham BL, Voloshin D, Wan M, Clarizio A, Upadhye S (2022). Care of sexual and gender minorities in the emergency department: a scoping review. Ann Emerg Med.

